# Thoracoscopic plication of the membranous portion of crescent-type tracheobronchomalacia in an elderly patient: a case report

**DOI:** 10.1186/s40792-020-00831-y

**Published:** 2020-04-06

**Authors:** Ryusuke Machino, Tsutomu Tagawa

**Affiliations:** Department of Thoracic Surgery, National Hospital Organization Nagasaki Medical Center, 1001-1 Kubara, Omura, Nagasaki, 856-8562 Japan

**Keywords:** Plication, Tracheobronchomalacia, Thoracoscopy

## Abstract

**Background:**

It is presumed that tracheobronchomalacia in adults is caused by airway pressure-induced injury due to chronic cough related to pulmonary emphysema or chronic bronchitis. Commonly, a posterolateral approach using stabilizing materials is the surgical technique of choice for treating tracheobronchomalacia. We report a case in which thoracoscopic plication of the membranous portion was performed instead of airway stent placement for tracheobronchomalacia in an elderly individual.

**Case presentation:**

An 87-year-old man who had been treated for bronchial asthma, pulmonary emphysema, and tracheobronchomalacia was admitted to our hospital with acute exacerbation of dyspnea. The patient underwent tracheal intubation, which was followed by tracheostomy 16 days later. Insertion of the tip of the adjustable-length tracheostomy tube to the end of the stenotic lesion enabled him to breathe spontaneously. However, conservative management failed due to recurrent pneumonia caused by the tracheobronchomalacia. Crescent-type tracheobronchomalacia (Johnson’s classification grade III) was diagnosed, and the main narrowed area of the trachea was assumed to be approximately 3–10 cm from the tracheal bifurcation. A thoracoscopic approach was selected because a posterolateral approach was considered too invasive considering the patient’s age and general condition. We placed eight stitches on the tracheal membranous portion and four stitches on the membranous portion of the right main bronchus, using the horizontal mattress suture technique. The use of foreign materials was avoided because meropenem-resistant *Pseudomonas aeruginosa* was cultured in a tracheal specimen. Immediately after the operation, the expiratory airway stenosis improved, and subsequently, spontaneous ventilation was possible using a normal type of tracheostomy tube instead of an adjustable-length tracheostomy tube.

**Conclusions:**

Tracheobronchomalacia is not a rare condition in patients with chronic obstructive pulmonary disease. The thoracoscopic approach is less invasive than the posterolateral approach and is suitable in cases that are otherwise refractory to medical treatment. We believe that thoracoscopy may be a useful treatment option in cases where conservative treatment is not appropriate.

## Background

Tracheobronchomalacia in adults is believed to be caused by airway pressure-induced injuries due to chronic cough secondary to pulmonary emphysema or chronic bronchitis [1, 2]. Masaoka et al. classified the disease into the following three types based on the etiology: pediatric, adult, and secondary types [3]. In terms of form, tracheobronchomalacia is classified as the saber-sheath type and the crescent-moon type (crescent-type). In the former type, the lateral diameter is shortened during exhalation, and both sidewalls are closed, whereas in the latter type, the transverse diameter expands during exhalation and the membranous portion is closed. The Johnson’s classification divides airway lumen obstruction during coughing into the following four grades: grade I (50% or less), grade II (50-75%), grade III (75-100%), and grade IV (complete obstruction during coughing) [4]. Although conservative therapy is the mainstay of treatment for this disease, surgical treatment is considered in severe cases [5]. We report a case in which thoracoscopic plication of the membranous portion was performed for tracheobronchomalacia, instead of using foreign substances, in an elderly individual.

## Case presentation

An 87-year-old man who had been treated for bronchial asthma, pulmonary emphysema, and tracheobronchomalacia (modified British Medical Research Council questionnaire grade III) at the department of internal medicine in our hospital was admitted for acute exacerbation of dyspnea. The patient’s pulmonary function before hospitalization was as follows: vital capacity (VC), 1200 mL; %VC, 46%; forced expiratory volume in one second (FEV1. 0), 500 mL; and FEV1. 0%, 45.7%.

The patient underwent tracheal intubation, followed by tracheostomy 16 days later. Insertion of the tip of an adjustable-length tracheostomy tube to the end of the stenotic part enabled him to breathe spontaneously. However, it was difficult to continue conservative management because he repeatedly suffered from pneumonia with meropenem (MEPM)-resistant *Pseudomonas aeruginos*a. Sixty-five days after admission, he was referred to our department for surgical treatment.

**Fig. 1:**
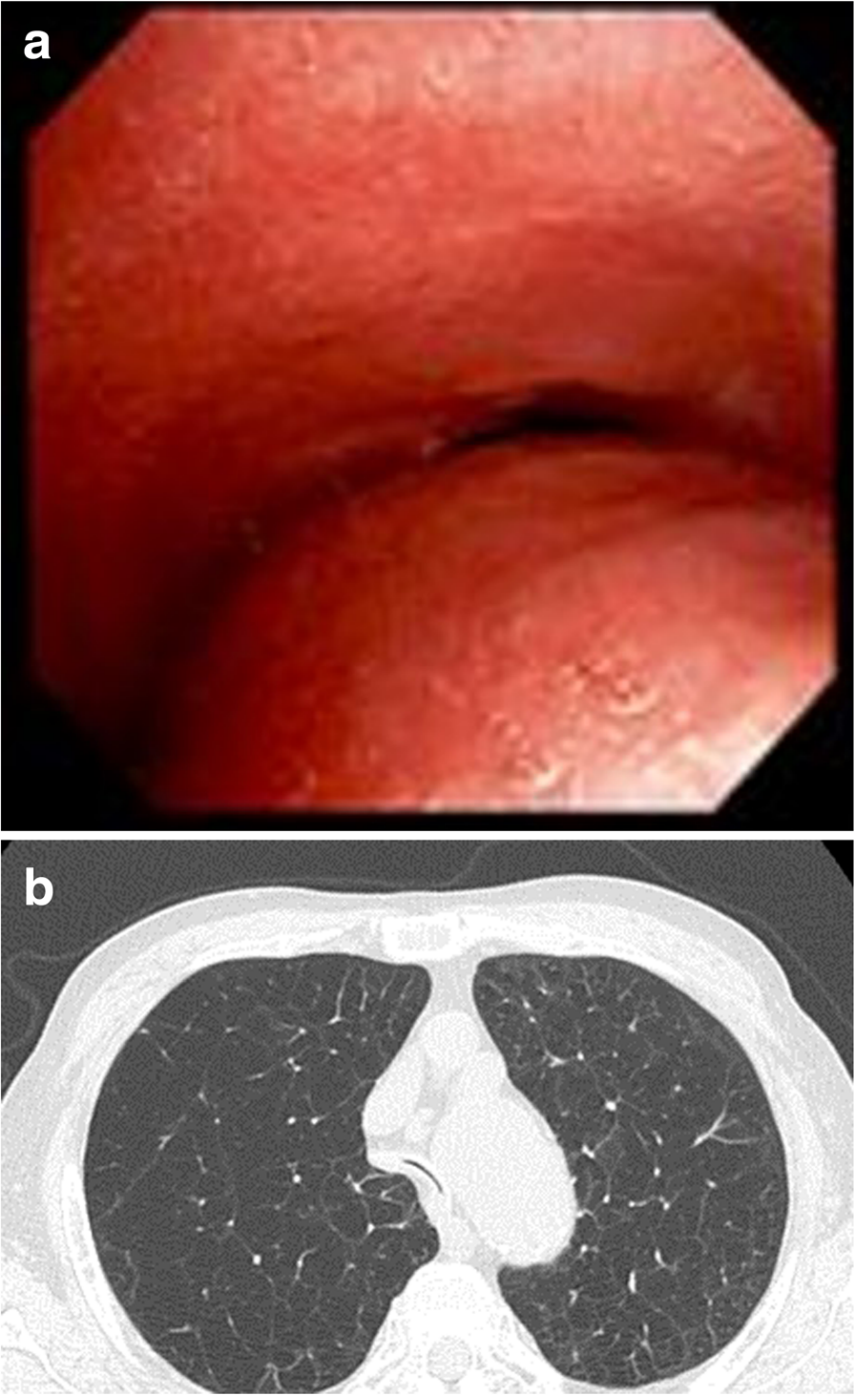
Bronchoscopy and chest computed tomography (CT). **a**. Bronchoscopic examination showing occlusion during coughing. **b** Chest CT revealing stenosis ranging from the trachea to the right main bronchus

The trachea was completely occluded during coughing. The crescent-type of tracheobronchomalacia (Johnson’s classification grade III) was also diagnosed based on the findings of a bronchoscopic examination (Fig. 1a). Chest computed tomography (CT) revealed stenosis ranging from the trachea to the main bronchus (Fig. 1b). However, the patient could breathe spontaneously when an adjustable-length tracheal tube was inserted. Therefore, the main narrowed area of the airway was assumed to be approximately 3-10 cm from the tracheal bifurcation.

**Fig. 2:**
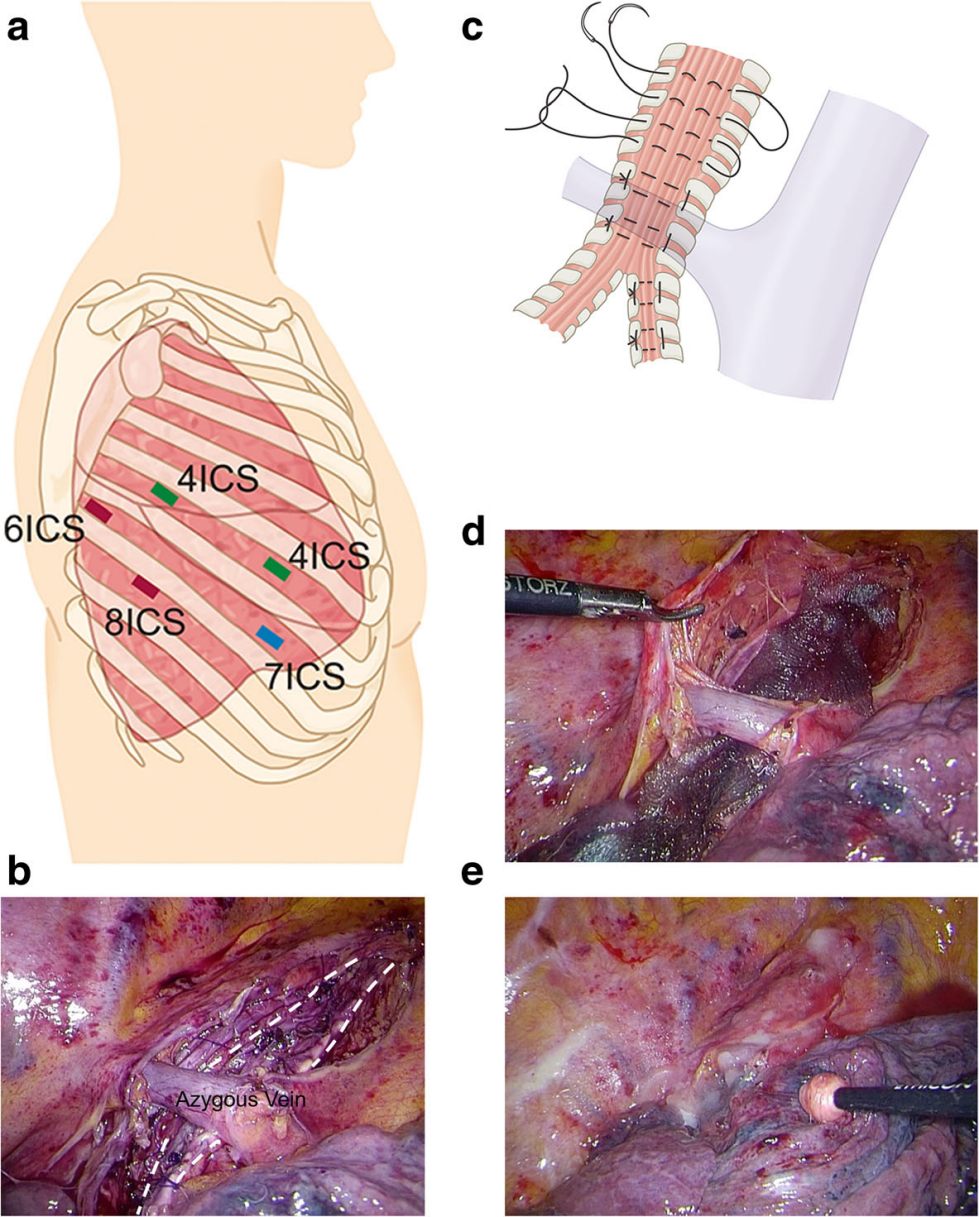
Surgical technique. **a** Schema of port sites in this surgery. **b** Plicated tracheal and right main bronchial membranous portion using the horizontal mattress suture technique with 4-0 PDS double-ended needles (within dotted line). **c** Schema of the plication technique. **d** Polyglycolic acid sheets on the sutured portion, fixed with fibrin glue. **e** The plicated portion covered with the mediastinal pleura

A posterolateral approach is commonly used for the plication or external fixation of the membranous portion in tracheobronchomalacia. In this case, we chose the thoracoscopic approach because the posterolateral approach was too invasive, considering the patient’s age and general condition. He underwent thoracoscopic surgery with five ports (Fig. 2a). A satisfactory observation field was attained under differential lung ventilation with a double-lumen tube and carbon dioxide insufflation to an intrathoracic pressure of 5 mmHg. We exposed the trachea and the right main bronchus just below the right subclavian artery. Thereafter, we placed eight sutures on the tracheal membranous portion and four sutures on the right main bronchial membranous portion, using the horizontal mattress suture technique with 4-0 PDS double-ended needles (Fig. 2b, c). The trachea and the right main bronchus were plicated by pulling up the loosened membranous portion to avoid damage to the tracheal tube, and the size of the trachea was matched with the size of the tracheal tube. The left main bronchus was not sutured. Additionally, we attached polyglycolic acid (PGA) sheets on the sutured portion using fibrin glue (Fig. 2d) and the mediastinal pleura (Fig. 2e) without suturing. We avoided the use of foreign materials as much as possible because MEPM-resistant *Pseudomonas aeruginosa* was cultured from a sample taken from the inside of the trachea. Immediately after the operation, the expiratory airway stenosis improved, and subsequently, spontaneous ventilation became possible with a normal type of tracheostomy tube instead of an adjustable-length tracheostomy tube. The severity of tracheomalacia improved to less than grade I in the Johnson’s classification.

**Fig. 3:**
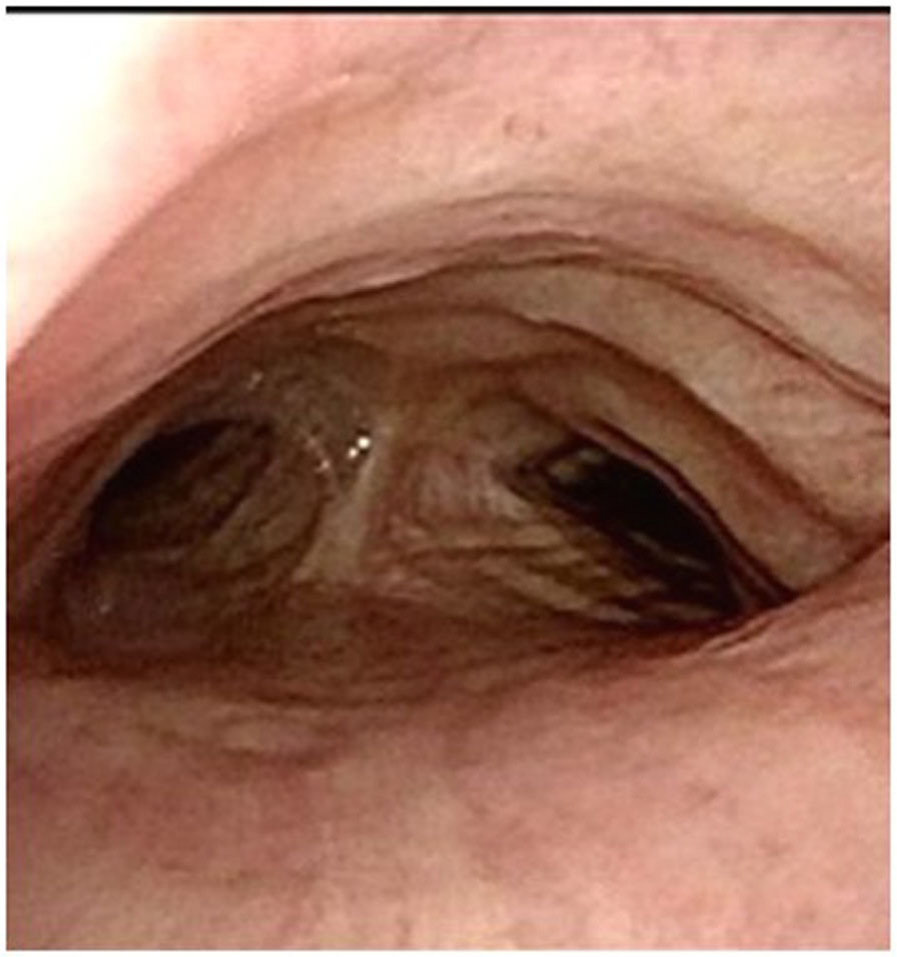
Postoperative bronchoscopy. The area that had been constricted preoperatively remains constricted at 6 months postoperatively

**Fig. 4:**
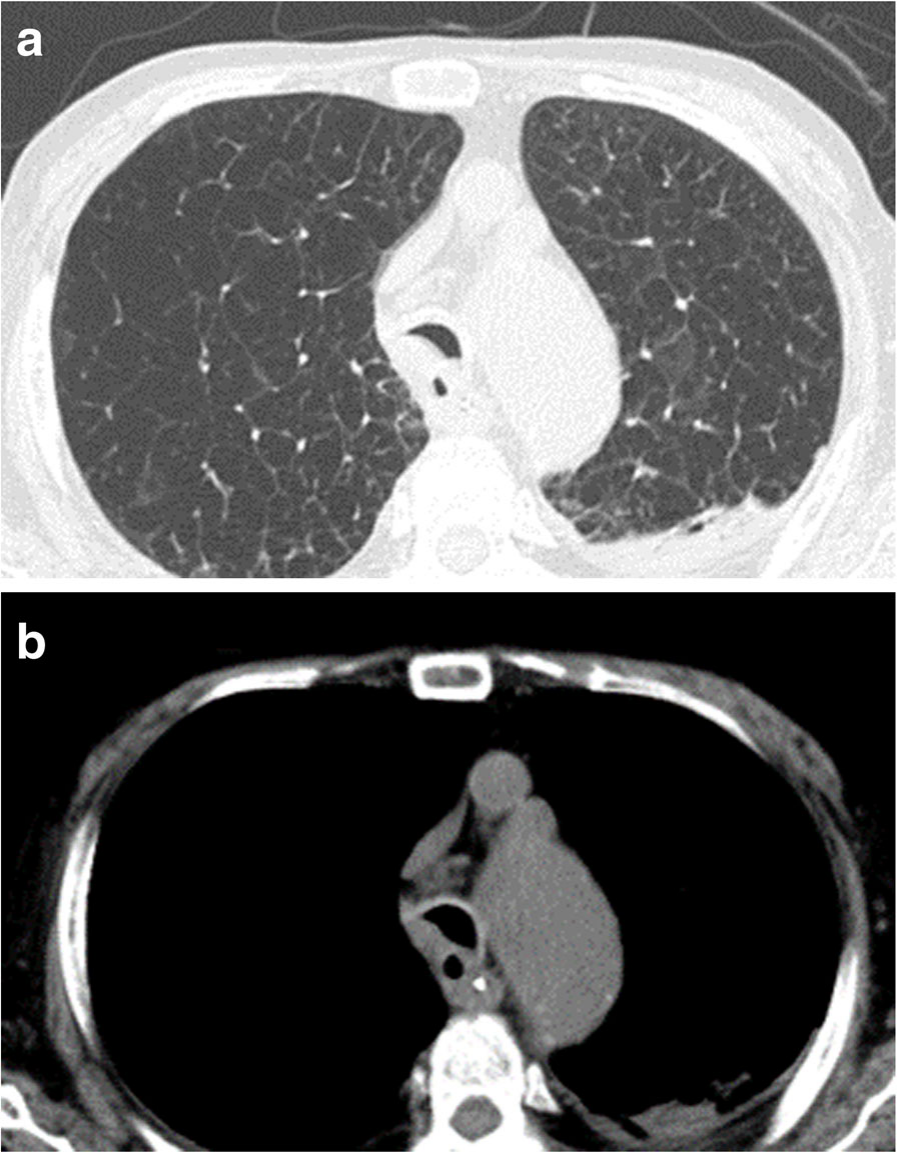
Postoperative chest computed tomography (CT). **a** Expansion of the stenotic area on chest CT at 6 months postoperatively. **b** Scars covering the dorsal side of the membranous portion of the bronchial tube at 6 months postoperatively

The patient needed long-term rehabilitation; however, he did not experience any difficulties in breathing, and the pneumonia was successfully treated. The patient was discharged on postoperative day 126 with a normal tracheostomy tube in situ. Bronchoscopy revealed that a preoperatively constricted area was present at 6 months after the operation (Fig. 3). The stenotic area was found to have expanded on chest CT. Despite avoiding the use of foreign materials, such as a mesh, the dorsal side of the membranous part was covered with a thickened scar tissue (Fig. 4a, b).

## Discussion

We reported a case in which thoracoscopic plication of the membranous portion was performed instead of the use of foreign substances for tracheobronchomalacia in an elderly individual.

For tracheobronchial extracorporeal fixation, Nissen et al. described an operation using autologous ribs [6], and Herzog et al. described an operation using the rectus abdominal muscle sheath [7]. Subsequently, Wright et al. reported on extra-membranous external fixation using a polypropylene mesh [8. 9]. In this technique, in addition to direct fixation of the membranous portion with a polypropylene mesh, the scar tissue grows, and the polypropylene mesh is integrated into the membranous portion to increase its stability and to maintain airway lumen patency [5]. Thus, fixation of the membranous portion with stabilizing materials, such as polypropylene meshes [8, 9] or polytetrafluoroethylene sheets [10], is the most common approach in recent years. In our case, after applying PGA sheets on the membranous portion, the area around the membranous portion was covered with thick scar tissue at 6 months after the operation although stabilizing materials were not used, and this also led to the stability of the membranous portion. The fixation was adequate, and restenosis was not observed. The suturing may cause restenosis long term; however, it is considered appropriate when performing lifesaving surgery in the elderly.

Indications for surgical treatment of tracheobronchomalacia include the absence of irreversible respiratory disorders, such as chronic obstructive pulmonary disease (COPD), and any other underlying disease that could complicate surgery. However, Ernst et al. reported the effectiveness of extra-membranous fusion using a polypropylene mesh for tracheobronchomalacia associated with severe emphysema. According to their report, airway stabilization is indicated in patients whose respiratory condition is improved by placing a silicone stent [11]. In the present case, assuming that the adjustable-length tracheostomy tube was equivalent to stent placement, it would have been considered an indication for surgery, because spontaneous breathing was possible by the placement of the adjustable-length tube.

In addition, this procedure is commonly performed using a conventional posterolateral approach. Most reports on this approach indicate that foreign materials such as polypropylene meshes [8, 9] are attached to the membranous portion. We aimed to reduce the invasiveness of the operation compared to that of the conventional posterolateral approach by performing it under thoracoscopy. The outcome of this less invasive, safer approach suggests that more patients can undergo this surgery. Additionally, we chose to avoid the use of foreign materials because MEPM-resistant *Pseudomonas aeruginosa* had been detected in the central airway. Hence, we decided to plicate the membranous portion with horizontal mattress sutures using absorbent threads at both ends of the needle and use only PGA sheets and fibrin glue for fixation. This method was adopted from the report by Masaoka et al. and modified [3]. We used horizontal mattress sutures in order to avoid tissue damage as much as possible and to increase the suture density to obtain a reliable forming effect. This method is advantageous because it avoids the use of foreign materials, and the procedure is simpler and easier to perform under thoracoscopy compared to establishing stabilization using a mesh. Although this method is considered useful for the crescent type of tracheobronchomalacia, it is necessary to fix the left and right cartilage parts for the saber-sheath type; hence, it is necessary to fix the trachea by a prosthesis.

First, we separated the lung ventilation by inserting an extra-long, single-lumen tube using an occlusion balloon, because the double-lumen tube was too large and could potentially be damaged by the suturing [9]. However, because the patient had COPD, the right lung was not sufficiently collapsed. Posterolateral thoracotomy allows for the surgery to be performed while isolating the lung that cannot be collapsed. In contrast, when performing thoracoscopic surgery, the single-lumen tube provides insufficient collapse of the lung. Thus, we provided unilateral lung ventilation with a double-lumen tube and carbon dioxide insufflation to create a sufficient operative field. Nevertheless, care must be taken to prevent damage to the tube-cuff during suturing. In addition, there have been several reports on azygous vein ligation and division [9, 12]. However, using the thoracoscopic field of view, the posterior aspect of the azygous vein could be sutured without tension.

However, because the cervical trachea cannot be visualized using a transthoracic approach, a tracheostomy or a permanent stent may be required when treating patients in whom malacia or stenosis extends to the cervical trachea.

## Conclusions

Plication of the membranous portion of crescent-type tracheobronchomalacia during thoracoscopic surgery results in similar outcomes compared to other approaches. The invasiveness of this procedure is much less than that of the posterolateral approach. We believe that thoracoscopy may be a useful treatment option in cases where conservative treatment is not appropriate.

## Data Availability

The datasets supporting the conclusions of this article are included within the article.

## Abbreviations

COPD: Chronic obstructive pulmonary disease
CT: Computed tomography
FEV1.0: Forced Expiratory Volume in one second
MEPM: Meropenem
PGA: Polyglycolic acid
VC: Vital capacity
